# New insights into controlling horticultural crop quality deterioration caused by cold stress: epigenetic modification

**DOI:** 10.1093/hr/uhaf326

**Published:** 2025-11-27

**Authors:** Xiaodong Fu, Fujun Li, Yanan Li, Xiaoan Li, Xinhua Zhang, Zienab F R Ahmed

**Affiliations:** College of Agricultural Engineering and Food Science, Shandong University of Technology, Zibo 255000, Shandong, PR China; College of Agricultural Engineering and Food Science, Shandong University of Technology, Zibo 255000, Shandong, PR China; College of Agricultural Engineering and Food Science, Shandong University of Technology, Zibo 255000, Shandong, PR China; College of Agricultural Engineering and Food Science, Shandong University of Technology, Zibo 255000, Shandong, PR China; College of Agricultural Engineering and Food Science, Shandong University of Technology, Zibo 255000, Shandong, PR China; Integrative Agriculture Department, College of Agriculture and Veterinary Medicine, United Arab Emirates University, Al Ain 15551, United Arab Emirates

## Abstract

Low-temperature environments cause chilling injury in horticultural crops and accelerate quality deterioration after rewarming, which is closely related to epigenetic modifications. Epigenetic regulation is widely involved in various aspects of cold responses in horticultural crops, including the expression of cold-tolerant proteins, dynamic changes in cell membranes, energy metabolism, and reactive oxygen species metabolism. With the emergence and development of new scientific technologies, uncovering the secrets of epigenetic regulation in horticultural crop quality is becoming possible. Therefore, this paper reviews the types, roles, and potential mechanisms of epigenetic modifications involved in cold stress responses in horticultural crops, summarizes the dynamic changes and effects of exogenous treatments on epigenetic modifications, and discusses the feasibility of new editing technologies in epigenetic research and applications. This review aims to elucidate the complex regulatory mechanisms of epigenetic control in cold responses in horticultural crops, providing a theoretical foundation for developing novel strategies to control quality decline in horticultural crops.

## Introduction

Low temperature is a critical environmental factor limiting the geographical distribution of horticultural crops. Extremely low temperatures can trigger chilling injury (CI) in horticultural crops and cause rapid quality deterioration [1]. Additionally, during postharvest storage and distribution of horticultural byproducts, low-temperature storage is a primary strategy to delay senescence and quality deterioration, although prolonged cold storage can also cause quality decline in cold-sensitive horticultural products [2]. However, plants have evolved complex and effective response strategies to adapt to low-temperature environments. These adaptive mechanisms encompass a range of molecular responses, such as C repeat binding factor (CBF) expression inducer 1 (ICE1)-CBF-cold regulated (COR) pathway, calcium (Ca^2+^) signaling cascades, membrane metabolism, carbohydrate metabolism, lipid metabolism, photosynthesis, and antioxidant systems (including enzymes and compounds) [2, 3]. Despite significant research efforts to elucidate metabolic pathways for controlling quality deterioration of horticultural crops caused by low temperature, the complete regulatory network is still not fully understood.

Epigenetics refers to a genetic mechanism that mediates transcriptional reprogramming influenced by environmental factors, without changes to the DNA sequence. This mechanism guides cells to regulate the expression of specific gene sets in a spatiotemporal manner, enabling responses to environmental cues and developmental demands [4]. Emerging research shows that environmental stresses, such as cold stress, can trigger epigenetic modifications, which are divided into transient and persistent states; most modifications are transient, dynamically regulating the expression of responsive genes through targeted modifications by a series of specific enzymes; persistent epigenetic modifications form ‘stress memory’ within chromatin in response to stress; after the stress ends, these modifications return to baseline levels, but upon re-exposure to the same stress, they rapidly regulate genes transcription to mount a more effective response [5]. These epigenetic modifications may be inherited across multiple plant generations as stable ‘stress memory’ [5]. Research on horticultural crops has shown that cold stress can strongly trigger transient and ‘stress memory’-associated epigenetic modifications [5–7]. For example, low temperature leads to flavor loss in tomato and peach fruits by altering the methylation levels of specific gene promoters [8, 9]. These epigenetic modifications broadly regulate metabolic pathways involved in cold stress responses, such as hormone signaling interactions and the accumulation of metabolites. Although a close association between epigenetics and cold stress responses in horticultural crops has been uncovered, the understanding of the specific regulatory mechanisms still remains in its early stages. Thus, to elucidate the regulatory mechanisms by which epigenetic modifications control CI in horticultural crops, this paper reviews the types and mechanisms of epigenetic modifications in cold stress responses, summarizes methods and effects of exogenous induction of epigenetic modifications in horticultural crops, discusses the feasibility of new technologies integrating epigenetic inheritance, and provides valuable perspectives for future research directions and applications, aiming to develop novel, efficient, and safe strategies for controlling CI in horticultural crops.

## Epigenetic modifications involved in cold stress responses in horticultural crops

### DNA methylation

**Figure 1:**
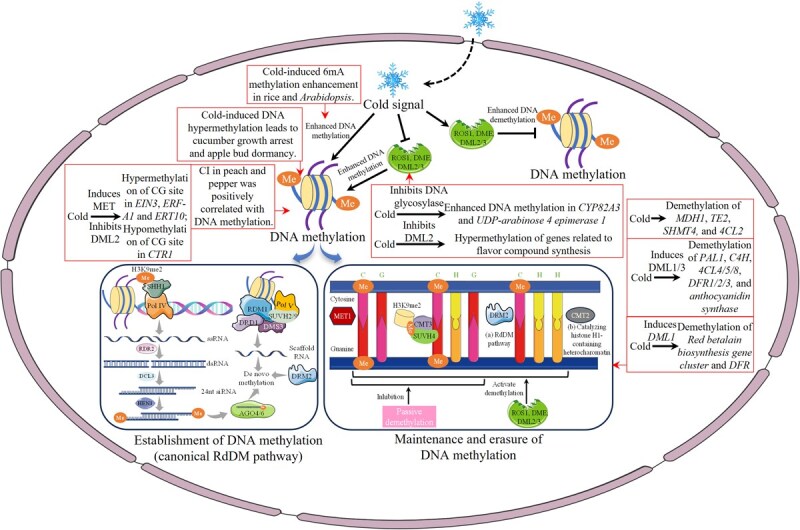
Establishment, maintenance and erasing of 5mC DNA methylation [13]. (1) Canonical RNA-directed DNA methylation (RdDM) pathway: Sawadee homeodomain homolog 1 (SHH1) and Pol IV generate single-stranded RNA (ssRNA), which is converted into double-stranded RNA (dsRNA) by RNA-dependent RNA polymerase 2 (RDR2), and then cleaved into 24 nt siRNA by the endonuclease Dicer-like protein 3 (DCL3). The mature 24 nt siRNA, methylated by Hua enhancer 1 (HEN1), is loaded onto the effector protein Argonaute (AGO) 4/6, pairs with scaffold RNA produced by the Pol V and Defective in RNA-directed DNA methylation 1-RNA-directed DNA Methylation 1-Defective in meristem silencing 3-suppressor of Variegation Homologous (DRD1-RDM1-DMS3-SUVH) protein complex, and recruits domain rearranged methylase 2 (DRM2) for *de novo* methylation. (2) Maintenance of DNA methylation: The maintenance of DNA methylation in plant follows the semiconservative replication principle, where pre-existing methylation modifications in the parental strand lead to new methylation modifications at corresponding symmetric (CG and CHG) sites in the daughter strand, while maintenance of the asymmetric (CHH) sites does not rely on semireserved replication, but follows a *de novo* methylation pathway. (3) Erasure of DNA methylation: DNA demethylation includes two mechanisms: active demethylation and passive demethylation. Active demethylation is catalyzed by specific reactions of DNA glycosylases and lyases, with repressor of silencing1 (ROS1), demeter (DME), demeter-like (DML)2, and DML3 responsible for DNA demethylation in different tissues [15]. Passive demethylation refers to the loss of methylation modifications on the daughter strand due to insufficient activity of DNA methyltransferases during DNA semiconservative replication, which inhibits the maintenance of symmetric site methylation. MET1: methyltransferase 1; CMT: chromethylase; MDH: mitochondrial malate dehydrogenase; TE: Ketoacyl-ACP thioesterase; SHMT: Serine hydroxymethyltransferase; DFR: dihydroflavonol 4-reductase; PAL: phenylalanine ammonia lyase; C4H: cinnamate 4-hydroxylase; 4CL: 4-coumarate: CoA ligase.

DNA methylation is the most extensively studied epigenetic modification in plants [10]. In plants, DNA methylation is primarily achieved by adding a methyl group to the fifth carbon atom of cytosine (5-methylcytosine [5mC]) or the sixth nitrogen atom of adenine (N6-methyladenine [6 mA]) [11]. Among these, 5mC predominantly occurs in the sequence contexts of CG, CHG, and CHH (H = A/T/C); 6 mA mainly occurs in the sequence contexts of ANYGA and GAGG, which is conserved in plants [11, 12]. In horticultural crops, DNA methylation is dynamically regulated by methyltransferases and demethylases. The methylation process of 5mC mainly includes three states: *de novo* methylation, maintenance methylation, and demethylation, the detailed mechanisms are shown in Fig. 1 [13]. The 5mC primarily occurs in gene body regions and, to a lesser extent, in promoter regions, generally suppressing gene expression. This suppression may arise from two possible reasons: (i) 5mC modifications in promoter and enhancer regions may interfere with the binding of transcription factors to their target genes and (ii) 5mC-modified sites may attract repressive histone modifications, leading to chromatin compaction [13, 14]. Furthermore, 5mC modifications have been observed to activate gene expression in certain instances [13]. Unlike 5mC, 6 mA methylation is less common in plants and plays distinct roles in gene bodies (primarily involved in gene activation) and promoters (primarily involved in gene repression) [14]. Additionally, the full mechanistic system of 6 mA methylation in plants is not yet fully understood [11]. Nevertheless, current research indicates that external factors like cold stress are key drivers of DNA methylation changes in horticultural crops. Cold-mediated dynamic DNA methylation not only regulates flowering cycles through vernalization in plants [11], but also plays a role in quality regulation of cold response in horticultural crops.

**Table 1:** The growth status of horticultural crops that may be epigenetically related at low temperatures.

Crops	Chilling injury	Epigenetic type	Refs.
Peach	Loss of softening ability; internal browning	CHH site DNA methylation	[16]
	Powdery texture	Increased DNA methylation of *CYP82A3* and *UDP-arabinose 4-epimerase 1*	[17]
	Loss of flavor substances	Increased DNA methylation of *ACS1*, *AAT1*, *TPS3* and *MADS2*	[9, 27]
	Accumulate anthocyanins	Demethylation of *PAL1*, *cinnamate 4-hydroxylase*, *4-coumarate: CoA ligase 4/5/8*, *DFR1/2/3*	[24, 26]
Tomato	Loss of flavor substances	Increased DNA methylation of *RIN*, *NOR*, and *CNR*	[8]
	Delay of maturation	Increased DNA methylation of *ethylene insensitive 3*, *ethylene responsive factor-A1*, and *ethylene-responsive transcripts*	[22, 23]
	Enhancing survival rate	SWI/SNF complex BCL7/BCL7A interacting domain inhibits WRKY34	[51]
	Fruit ripening inhibition; surface depression	Accumulation of SlCHD9-like; transcriptional inhibition of *PICKLE*; differential expression of SWI/SNF family genes	[53]
	Affecting the ripening and quality of the fruit	m^6^A methylation of *HSP70-binding protein* and *WRKY81*	[63]
Pepper	Growth inhibition	Increased DNA methylation	[18]
	Leaves wither; growth state is inhibited	Upregulation of *m^6^A methyltransferase* and *demethylase*	[65]
Cucumber	Root growth stagnation	Genomic high methylation	[19]
Brassica rapa	Improving growth performance	Demethylation of *mitochondrial malate dehydrogenase1*, *Ketoacyl-ACP Thioesterase2*, *Serine Hydroxymethyltransferase4 and 4-Coumarate-CoA Ligase2*	[20]
Apple	Bud dormancy	High DNA methylation	[21]
	Flower drop and failure of fruit setting.	Deacetylation of MdABI1	[37]
Blood orange	Accumulate anthocyanins	Demethylation of *Ruby* and *DFR*	[25]
Banana	Browning	Increased acetylation of *ω-3 fatty acid desaturases*	[36]
Melon	Growth inhibition; deterioration of fruit quality	Increased acetylation of *MELO3C034813.2* and *MELO3C034814.2*	[38]
Strawberry	Coloring failure; reduction of flavor substances	H3K27me3 modification of the cold response gene	[42]
Potato	Accumulation of reducing sugars	Bivalent histone H3K4me3-H3K27me3 modifications	[45]
Mango	Ensure the normal growth of seedlings	3’UTR-enriched m^6^A methylation in mRNAs	[64]
Cassava	Enhance cold resistance	lncRNA-CRIR1 recruits methyltransferases or demethylases	[76]

Specifically, cells in horticultural crops sense cold signals and facilitate dynamic methylation regulation via associated enzymes, modulating the expression of genes in related metabolic pathways to respond to cold stress. Low-temperature-mediated dynamic methylation extensively affects CI regulation, oxidative metabolism, flowering, dormancy, ripening, flavor, and nutrient accumulation in horticultural crops. Firstly, cold conditions typically lead to an overall rise in DNA methylation in horticultural crops, which often exacerbates CI in crops. For example, postharvest peach fruit treated with gradient low temperatures (0, 5, 8, 12, and 16°C) exhibited overall higher levels of genome-wide DNA methylation, with CHH site methylation showing a strong positive correlation with loss of softening ability and internal browning [16]. Certain CI symptoms, such as the powdery texture observed in peach flesh, which may be due to cold treatment suppressing the expression of DNA glycosylase, leading to increased methylation of genes such as *CYP82A3* and *UDP-arabinose 4 epimerase 1* [17]. The symptom of CI in tomato and certain peach fruit is characterized by a reduction in flavor substances, this phenomenon occurs as low temperatures inhibit the expression of *demeter-like* (*DML*) genes, resulting in hypermethylation of the promoters or gene bodies of key volatile synthesis genes, such as *RIPENING INHIBITOR* (*RIN*), *NONRIPENING* (*NOR*), and *COLORLESS NONRIPENING* (*CNR*) in tomato, and *ACC synthase 1*, *alcohol acyltransferase 1*, and *terpene synthase 3* in peach, which subsequently suppresses their expression and impairs flavor development [8, 9]. This could be due to high methylation inhibiting the expression of genes associated with cold tolerance.

Secondly, cold-mediated DNA methylation affects the growth status of horticultural crops, including growth, dormancy and flowering. For instance, where under the same low-temperature treatment, both cold-tolerant and cold-sensitive peppers showed significantly increased DNA methylation ratios; however, the fully methylated DNA ratio in cold-tolerant peppers was significantly lower than in cold-sensitive peppers, accompanied by higher antioxidant enzyme activity, proline content, and lower growth inhibition [18]. 2.5°C treatment of cucumber radicles resulted in genomic high methylation and immediate growth arrest, with only 18.6% recovery of radicle growth after rewarming compared to untreated group [19]. Cold acclimation triggered locus-specific demethylation of key metabolic genes (*mitochondrial malate dehydrogenase1*, *Ketoacyl-ACP Thioesterase2*, *Serine Hydroxymethyltransferase4* and *4-Coumarate-CoA Ligase2*) in *Brassica rapa*, coordinately upregulating their expression and substantially improving growth performance [20]. Dormancy in apple buds also showed significant correlation with cold-induced DNA hypermethylation [21].

Additionally, cold-mediated DNA methylation can regulate horticultural crop quality formation, such as delaying ripening and nutrient accumulation. For instance, 5°C treatment of postharvest green tomato fruit significantly induced DNA *de novo* methylation and demethylation but inhibited the demethylation process of ripening-related genes, leading to delayed ripening of postharvest green fruit; after returning to room temperature, more genes underwent demethylation, restoring fruit ripening; however, not all parts of the fruit experienced demethylation during rewarming, leading to low-temperature-induced quality deterioration [22]. Specifically, low temperatures significantly induced the activity of methyltransferases and inhibited the activity of demethylase (DML2) in tomato fruit, leading to increased CG site DNA methylation levels in ethylene signaling-related genes *ethylene insensitive 3*, *ethylene responsive factor-A1*, and *ethylene-responsive transcripts 10*, and decreased CG site DNA methylation levels in the negative regulator *constitutive triple response 1*, thereby inhibiting tomato ripening [23]. In addition, anthocyanins serve as crucial antioxidants in fruit and play a significant role in determining fruit quality. For example, in peach fruit, the promoters of anthocyanin biosynthesis-related genes *phenylalanine ammonia lyase* (*PAL*) *1*, *cinnamate 4-hydroxylase*, *4-coumarate: CoA ligase 4/5/8*, *dihydroflavonol 4-reductase* (*DFR*) *1/2/3*, and *anthocyanidin synthase* exhibited the lowest methylation levels and highest transcriptional activity in the 0°C and 16°C groups, which may be closely related to DNA demethylases PpDML1 and PpDML3 [24]. In blood orange fruit, low temperatures were also found to significantly induce the transcription of DML1 in high-pigment regions, mediating DNA demethylation and transcriptional upregulation of the *Ruby* and *DFR* (anthocyanin synthesis key genes) promoters, promoting anthocyanin accumulation [25]. Therefore, horticultural crops exposed to low temperatures may activate certain cold-tolerance-related pathways and temporarily inhibit the metabolism of cold-sensitive pathways like ripening, mediated by dynamic DNA methylation regulation. If the dynamic changes of DNA methylation can be controlled, it would be possible to manage CI and quality deterioration in horticultural crops during cold storage. Further research has found that many exogenous treatments can partially achieve this goal. For example, melatonin treatment enhanced the activity of methyl esterase and demethylase in peach fruit at 4°C, leading to methylation of *polyphenol oxidase* (*PPO*) and *peroxidase* (*POD*) and demethylation of *PpPAL* (these modifications all occurred at the CG site), thereby upregulating *PpPAL* transcription and suppressing *PpPPO* and *PpPOD* transcription, promoting phenolic accumulation to inhibit browning [26]. Methyl jasmonate treatment can activate the ripening and aroma compound synthesis of peach fruit suppressed at 4°C by inducing demethylation of ethylene synthesis gene promoters, thereby restoring the ripening process of fruit under low temperatures [27]. Nitric oxide (NO) and the methylation inhibitor GSK343 also regulate CI in peach fruit and flowering process in litchi under low temperature conditions by controlling the activity of methylation-modifying enzymes [28, 29].

In summary, cold triggers dynamic DNA methylation changes in horticultural crops, where DNA hypermethylation typically shows positive correlation with increased chilling sensitivity and quality degradation (e.g. browning and flavor loss). Among these, CHH site methylation is closely related to CI, while ethylene signaling transduction and phenolic metabolism are primarily influenced by CG site methylation. The application of melatonin, MeJA, and others can reduce the methylation levels of cold-tolerance-related genes by inhibiting methyltransferases or promoting demethylase activity, thereby enhancing their expression and the cold tolerance of fruit. Nevertheless, the current research landscape is dominated by phenotype association studies combining DNA methylomics and transcriptomics, with a significant lack of functional validation for methylation-modifying enzymes and research on site-specific epigenetic editing—key aspects for elucidating the epigenetic mechanisms governing cold tolerance.

### Histone modification

Histone modifications are the processes of post-translational modification (PTMs), including methylation, acetylation, phosphorylation, and ubiquitination, that occur at the core regions of histones or the N-terminal tails extending from nucleosomes [30]. These modifications are regulated by low temperatures and can influence the interactions between histones and DNA or other proteins, thereby affecting chromatin structure [31]. Similar to PTMs of other proteins, all histone modifications are reversible, and specific modification patterns of histones may lead to unique transcriptional responses in horticultural crops under cold stress, providing flexible and precise strategies for coping with cold stress [32, 33].

**Figure 2:**
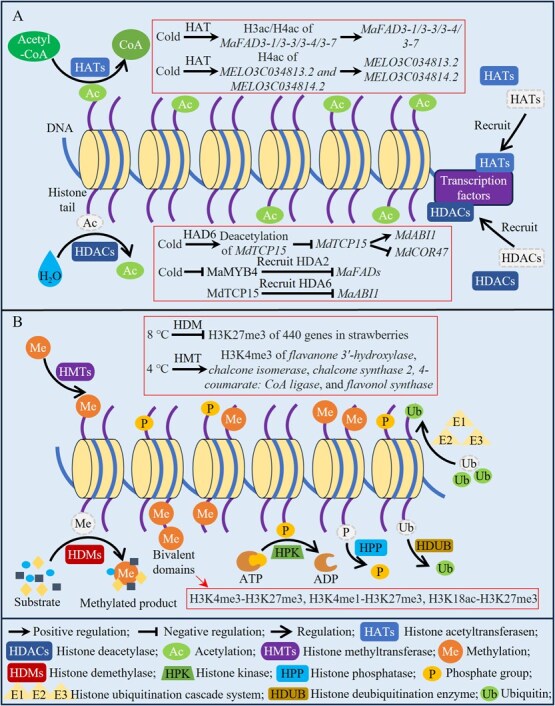
Histone modification mechanism involved in cold regulation of horticultural crops.

Among them, (I) acetylation: histone acetylation involves the transfer of an acetyl group from acetyl-CoA to the ε-amino group of lysine side chains by histone acetyltransferases, with removal mediated by histone deacetylases [34]. These histone acetylation-modifying enzymes respond to low-temperature signals, thereby regulating histone acetylation levels (Fig. 2A). For example, 37 *histone acetyltransferases* have been identified in citrus fruit, most of which contain low-temperature response elements (LTRs) in their gene promoters [35]. In banana fruit, MaMYB4 can physically interact with histone deacetylase (HDA)2, recruiting HDA2 to the promoters of *ω-3 fatty acid desaturases* (*FADs*) to suppress their acetylation levels; however, low temperatures inhibit MaMYB4 activity to activate the expression of target genes, subsequently suppressing browning by modulating the ratio of saturated to unsaturated fatty acids [36]. In apples, the transcription factor TEOSINTE BRANCHED1/CYCLOIDEA/PCF (TCP) 15 targets and activates the cold stress negative regulator *Abscisic acid-insensitive* (*ABI*) *1*, but under low temperature conditions, MdTCP15 recruits MdHDA6 to the *MdABI1* promoter, leading to deacetylation of *MdABI1* and inhibition of its transcriptional levels, thereby reducing low-temperature-induced flower drop and fruit set failure [37]. Further studies revealed that histone acetylation in horticultural crops typically neutralizes the positive charge on histone tails, reducing their affinity for negatively charged DNA and increasing DNA accessibility; this activates transcriptional regulation, whereas deacetylation generally suppresses transcriptional regulation [33]. For example, during cold stress in banana fruit, low temperatures induce increased acetylation levels of histones H3 and H4 (H3ac/H4ac) in the promoters of FADs-related genes (*MaFAD3-1*/*3-3*/*3-4*/*3-7*), enhancing the transcription of *MaFADs* and the conversion of linoleic acid to α-linolenic acid, which maintains membrane integrity under cold stress [36]. Through metabolomic and transcriptomic analyses of cold-tolerant melon varieties, the gene *eleven-nineteen lysine-rich leukemia-associated factor7*, encoding a nucleosome acetyltransferase of the H4 complex, was identified; it may control sugar and organic acid metabolism by modulating acetylation levels of histone H4 genes (*MELO3C034813.2* and *MELO3C034814.2*), thereby alleviating the low temperature induced growth inhibition and fruit quality deterioration [38]. In apples, MdTCP15 not only targets and activates *MdABI1* but also represses the expression of cold stress positive regulator *MdCOR47*; MdHDA6 inhibits its expression by deacetylating *MdTCP15*, thereby enhancing cold tolerance [37]. In addition, similar acetylation patterns were observed in the expression of other cold resistance-related genes, including the ICE1-CBF-COR pathway and reactive oxygen species regulation [32, 39].

(II) Methylation: histone methylation primarily relies on histone methyltransferases and histone demethylases to dynamically regulate the addition and removal of methyl groups on lysine and arginine residues (Fig. 2B) [40]. Unlike acetylation, histone methylation in horticultural crops does not affect the charge of histones, and its effects vary depending on the modification site and the number of modifications, with these sites are highly conserved in eukaryotes [41]. Histone methylation primarily occurs on lysine and arginine residues of H3 and H4; among these, trimethylation of lysine 27 on histone 3 (H3K27me3), H3K9, and symmetric dimethylation of arginine 3 on histone 4 (H4R3sme2) typically repress gene expression, while H3K4me3 and H3K36 modifications promote gene transcription [32, 42]. For example, strawberry fruit treated at 8°C exhibit failed coloration and a reduction in aroma compounds; chromatin immunoprecipitation sequencing revealed that this phenomenon is associated with the downregulation of 440 H3K27me3-modified genes, with a marked suppression of cold-responsive genes [42]. In fresh-cut pineapple stored at 4°C, caffeic acid treatment significantly increased H3K4me3 levels in key flavonoid biosynthesis genes (*flavanone 3′-hydroxylase*, *chalcone isomerase*, *chalcone synthase 2, 4-coumarate: CoA ligase*, and *flavonol synthase*), promoting their expression and flavonoid biosynthesis, thereby inhibiting quality deterioration such as flesh browning [43]. Additionally, the same gene promoter may simultaneously harbor both activating and repressive histone modifications, known as bivalent domains [44]. This modification increases the plasticity of gene expression, keeping genes in a ‘standby’ state, enabling them to respond to signals or stress stimuli and regulate gene expression (Fig. 2B) [44]. For example, in refrigerated potato tubers, 4°C induced the accumulation of reducing sugars and chromatin accessibility of active genes, which was closely associated with bivalent histone H3K4me3-H3K27me3 modifications [45]. H3K4me3-H3K27me3 is the most common bivalent modification in plants, and recent studies have identified bivalent modifications with varying methylation levels and types, such as H3K4me1-H3K27me3 in rapeseed and H3K18ac-H3K27me3 in *Arabidopsis* [46, 47]. Regrettably, the function of these atypical bivalent modifications in plant cold tolerance remains unexplored, marking a critical area for future investigation.

Beyond individual histone modifications, combinations of different histone modifications can serve as a code to regulate chromatin states, recognized and modulated by various complexes, thereby controlling the expression of cold-responsive genes [5, 47]. Under specific signals or stress stimuli, these histone codes are recognized and decoded by specific ‘histone code readers’, known as ‘stress memory’, which may be inherited across several generations; the diversity of histone variants and PTMs provides a vast array of possibilities for these codes, enabling precise epigenetic regulation of cold stress [5, 48]. Among these, the aforementioned bivalent histone modifications are one type of histone code.

In summary, beyond specific modification effects, histones can crosstalk with the same or other types of histone modifications to regulate gene expression, and the presence of histone codes offers further opportunities for epigenetic regulation of cold stress responses. However, current research on histone modifications in horticultural crops is limited, with existing studies primarily focusing on acetylation and methylation, while other modifications such as phosphorylation and ubiquitination remain unreported. Furthermore, deeper investigations into histone modification crosstalk and histone codes in the field of horticultural crops research are extremely rare.

### Chromatin remodeling factors and histone variants

**Figure 3:**
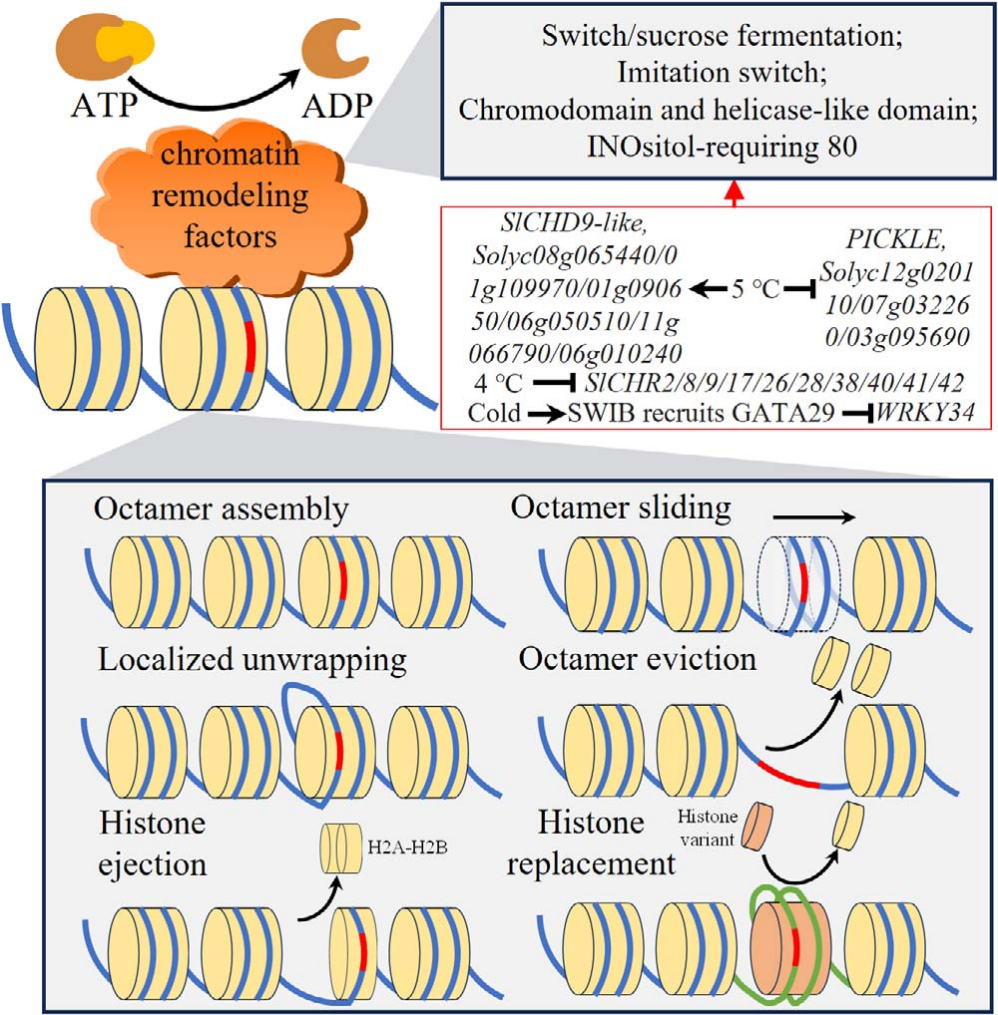
Chromatin remodeling factors and histone variants are involved in chromatin remodeling. SWIB: SWI/SNF complex BCL7/BCL7A interacting domain; CHD: helicase-like domain; CHR: Chromatin remodeling factor.

The regulation of remodeling factors and histone variants in chromatin remodeling is crucial for the regulatory process (Fig. 3). Chromatin is a nuclear complex composed of DNA and proteins, with nucleosomes as its basic unit, formed by DNA and the histone octamer (H2A, H2B, H3, and H4) that wraps around the DNA; nucleosomes are connected and stabilized by histone H1 [31]. Since chromatin remodeling is achieved through adenosine triphosphate (ATP)-dependent compression and decompression of nucleosomes, ATPases, as chromatin remodeling factors, play a crucial catalytic role in this process [49]. Chromatin remodeling complexes, composed of different ATPase subunits, are classified into four families: switch/sucrose fermentation (SWI/SNF), imitation switch (ISWI), chromodomain and helicase-like domain (CHD), and INOsitol-requiring 80 (INO80) [50]. Currently, the most studied ATPases in horticultural crops that respond to low temperatures are SWI/SNF ATPases and CHD ATPases. For example, low temperature induces the binding of the SWI/SNF complex BCL7/BCL7A interacting domain and transcription factor GATA29 to 60 bp InDel of cold tolerance negative regulator *WRKY34*, thereby suppressing WRKY34-mediated disruption of the CBF-COR pathway and enhancing cold stress survival rate in tomato seedlings [51]. In tomatoes, 45 SNF2 protein-coding genes (*Chromatin Remodeling Factor*, *SlCHRs*) have been identified, among which *SlCHR2/8/9/17/26/28/38/40/41/42* are primarily expressed in fruit and significantly suppressed at 4°C; these SNF2 family proteins can utilize ATP energy to alter chromatin structure and nucleosome positioning, thereby regulating gene expression to affect plant cold tolerance [52]. Transcriptomic analysis of tomato fruit showed that cold stress at 5°C induces significant differential expression of chromatin remodeling-related genes, with *SlCHD9-like* transcripts in the CHD family highly accumulated and the transcription of CHD3-type chromatin-remodeling factor *PICKLE* significantly suppressed; additionally, in the SWI/SNF family, 3 SNF2-related genes (*Solyc12g020110*, *Solyc07g032260*, and *Solyc03g095690*) were significantly downregulated, while 6 SNF2-related genes (*Solyc08g065440*, *Solyc01g109970*, *Solyc01g090650*, *Solyc06g050510*, *Solyc11g066790*, and *Solyc06g010240*) and 3 SWI/SNF residue genes (*Solyc01g109510*, *Solyc04g082760*, and *Solyc06g060120*) were significantly upregulated; these alterations may be associated with the inhibition of fruit ripening and surface pitting under low temperature [53]. Similarly, SWI/SNF and ISWI genes have also been found to regulate physiological processes under cold stress in crops such as pineapple, tomato, and *Arabidopsis* [53–55]. These results suggest that SWI/SNF-, CHD-, and ISWI-mediated chromatin remodeling may play a crucial role in the gene expression response to cold stress in horticultural crops.

The majority of histone genes are intronless; histones are synthesized during specific cycles and then deposited into DNA with the assistance of specific chaperone proteins and DNA polymerase [56]. In contrast, histone genes with introns are expressed in diverse forms through alternative splicing, referred to as histone variants [39]. These variants are expressed independently of the cell cycle and chromatin deposition, and their structural differences and varying DNA affinities may enable them to play a crucial role in cold stress-induced chromatin dynamic remodeling (Fig. 3) [39]. For instance, in *Arabidopsis*, the expression of most genes encoding histone variants is induced by cold stress [41]. And the deposition of H2A.Z markedly increases the plant’s responsiveness to temperature variations [57]. The transcriptional activation of flowering gene *FLOWERING LOCUS C* (*FLC*) in *Arabidopsis* requires the histone variant H2A.Z, and under prolonged cold conditions, FRIGIDA (FRI) directly regulates H2A.Z deposition to form a transcriptionally active chromatin structure, accelerating the FLC-mediated flowering process [58]. These variants, which replace canonical histones, may influence levels of DNA methylation and PTMs of histone during cold stress, thereby regulating the expression of cold-responsive genes [59]. Regrettably, research on how histone variants regulate cold responses in plants is still scarce, and such evidence has not yet been identified in horticultural species. Moreover, the flowering process mediated by FRI and FLC under cold conditions requires the involvement of the INO80-family SWI2/SNF2-Related 1 complex [58], which suggests from another perspective that the chromatin remodeling factor INO80 may participate in plant cold response by mediating histone variant deposition.

In summary, the coordinated regulation of chromatin remodelers and histone variants constitutes a pivotal determinant of chromatin-mediated transcriptional regulation in cold stress responses. However, current research has only established an association between chromatin remodelers and cold response in horticultural crops, while in-depth mechanistic investigations integrating gene editing with omics approaches remain scarce. Beyond this, there are more mysteries between cold stress and chromatin remodeling, such as how cold affects chromatin state attached to the nuclear membrane by altering its fluidity, how it modifies interactions between nucleic acids or between nucleic acids and proteins within chromatin, and how it influences protein folding and degradation in chromatin. These specific mechanisms require further investigation.

### RNA methylation

**Figure 4:**
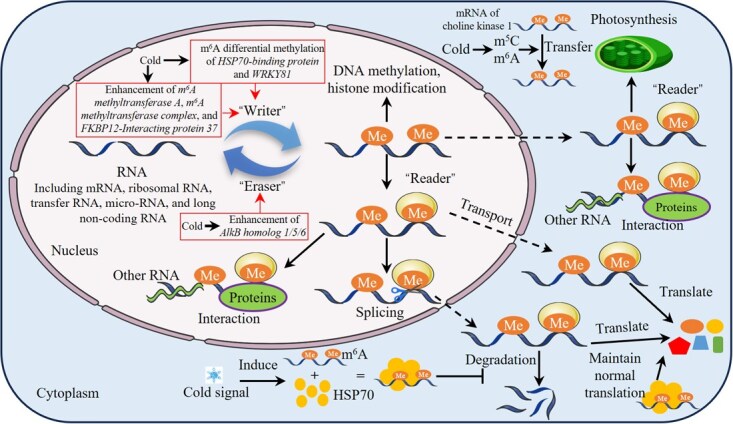
Regulation mechanism of RNA methylation in horticultural crops under cold stress.

Beyond DNA methylation, post-transcriptional RNA modifications serve as crucial epigenetic mechanisms in horticultural crops for responding to low temperatures. N6-methyladenosine (m^6^A) RNA methylation is the most prevalent RNA modification in plants, regulated by the ‘Writer (methyltransferases adding m^6^A modifications)-Eraser (demethylases removing m^6^A modifications)-Reader (recognizing m^6^A modifications)’ system, primarily controlling messenger RNA (mRNA) stability, splicing, transport, and translation (Fig. 4) [60]. m^6^A methylation is predominantly enriched at stop codons and 3′ untranslated (UTR) regions, a conserved pattern in plants [61]. Moreover, m^6^A modifications have been identified in ribosomal RNA, transfer RNA, micro-RNA (miRNA), and long noncoding RNA (lncRNA) [60].

The role of m^6^A methylation in regulating cold responses in horticultural crops has been extensively documented. For example, genome-wide analysis in litchi identified a large number of m^6^A -modified genes containing LTRs [62]. In cold-treated tomato fruit, differential m^6^A methylation primarily occurs on cold stress response factors such as *heat shock protein* (*HSP*)*70-binding protein* and *WRKY81*, as well as on transcripts of genes related to hormone metabolism, fruit texture, and redox reactions, which may influence fruit ripening and quality [63]. Under cold stress, mango seedlings exhibit 3′UTR-enriched m^6^A methylation in mRNAs; the m^6^A-modified mRNAs preferentially bind to HSP70 to form granular condensates, ensuring their stability and efficient translation, thereby supporting normal seedling growth [64]. Cold treatment also significantly induces expression of m^6^A methyltransferases (*methyltransferase A*, *m^6^A methyltransferase complex*, and *FKBP12-Interacting protein 37*) and demethylases (*AlkB homolog 1/5/6*) in pepper seedlings, which is associated with leaf wilting and suppressed growth under low temperature [65]. Furthermore, m^6^A methylation is involved in processes such as photosynthesis, DNA methylation, histone modifications, and chromatin dynamics under low-temperature stress [66, 67]. Specifically, m^6^A and 5mC mutually influence the expression of genes related to their modifications to adjust RNA/DNA methylation levels; m^6^A is involved in the recruitment and expression of histone-modifying enzymes and chromatin remodelers, all of which affect chromatin accessibility [67].

Based on current evidence, the response of m^6^A to low temperature appears to be faster than that of DNA methylation. As a rapid and reversible modification of pre-existing transcripts, m^6^A can instantaneously regulate mRNA stability, translational efficiency, and splicing [68, 69]; whereas DNA methylation primarily functions by altering chromatin state and transcriptional activity, typically corresponding to long-term or homeostatic responses [23, 24]. Most transcriptomic evidence also indicates that m^6^A methylation and DNA methylation are typically sampled at short-term (hours) and long-term (days) time points, respectively [9, 27, 65, 68]. However, this evidence remains speculative, and definitive conclusions require further experimental validation. Moreover, studies have found that the regulatory role of m^6^A methylation is not fixed but is closely related to m^6^A-modified genes and sites, specific recognition by ‘Reader’ proteins, dynamic RNA methylation, and environmental signals [69]. Besides m^6^A, m^5^C is also an important RNA methylation modification in plants. For instance, the mRNA of *CHOLINE KINASE 1* acts as an important long-distance signaling molecule in cold responses, and its mobility under low-temperature stress is crucial for enhancing the cold tolerance of cucumber (heterologous)/pumpkin, a process co-regulated by m^5^C and m^6^A modifications [70]. Yet, further studies are lacking, and research on RNA methylation modifications in horticultural crops remains a substantial area of unexplored territory.

### ncRNA

ncRNA represents a critical category in epigenetic regulation, modulating gene expression by engaging in and regulating various epigenetic modifications. ncRNA includes lncRNA and small ncRNA (sncRNA), and studies have shown that they are significantly affected by low-temperature stress [71–73]. Among these, lncRNA is involved in the processes of DNA methylation and demethylation. During the RNA-directed DNA methylation-mediated establishment of *de novo* DNA methylation, lncRNA serves as a scaffold to target specific genomic sequences [74]. Furthermore, after plant are exposed to cold stress, lncRNA not only targets cold-tolerance-related proteins but also regulates histone methylation and other modifications of downstream genes by recruiting histone-modifying enzymes, thereby modulating chromatin states to mediate the expression of cold-responsive genes [72, 75]. For instance, in cassava, the overexpression of lncRNA-CRIR1 significantly enhances cold tolerance, potentially achieved through the demethylation of promoters in genes associated with cold tolerance (including photosynthesis-related genes and transcription factors). The above process does not alter the expression of genes encoding methyltransferases or demethylases, suggesting it may function through the direct or indirect recruitment of these enzymes [76]. sncRNA is further divided into small interfering RNAs (siRNA) and miRNA [71]. siRNA, derived from double-stranded RNA, also participates in the establishment of *de novo* DNA methylation. As mentioned earlier, siRNA is loaded onto argonaute proteins, and through base pairing with scaffold RNA produced by Pol V and protein complexes, it recruits methyltransferases for *de novo* methylation modifications (Fig. 1) [13]. Mature miRNA can guide the RNA-induced silencing complex to target mRNAs of histone modification-related genes, ultimately leading to mRNA degradation or translational repression, thereby influencing the methylation state of the genome [74]. Additionally, miRNA can target lncRNA, potentially influencing the dynamic changes in lncRNA-mediated DNA methylation [77]. Although the roles of lncRNA, miRNA, and siRNA in regulating cold tolerance in horticultural crops and their mechanisms of participating in epigenetic modifications have been extensively demonstrated [13, 78], and their role in the epigenetic regulation of cold response has been verified in model plants such as *Arabidopsis* and rice, as well as in a few horticultural species, the current state of this field still requires large-scale exploration and in-depth mechanistic investigation.

## Application of epigenetic regulation in the control of CI

Emerging evidence indicates that epigenetic modifications in horticultural crops and other plant species function not merely as a passive ‘outcome’ of low-temperature exposure but also as an active ‘driver’ of chilling injury phenotypes. Various epigenetic regulatory undergo alterations under cold stress, inducing or amplifying observable pathological manifestations (Table 1); these epigenetic alterations have been demonstrated in several studies to exhibit causal or quasi-causal relationships with phenotypes (They can be alleviated or aggravated through experimental interventions.)

### Targeted epigenetic editing

Epigenetics is a deep regulatory mechanism in horticultural crops that controls gene expression in response to stress. Manipulating epigenetic mechanisms can greatly delay CI and the resulting quality decline in horticultural crops. The rapid advancement of modern gene-editing technologies, particularly the development of clustered regularly interspaced short palindromic repeats/endonuclease (CRISPR/Cas) systems, has made this concept feasible. Deactivated Cas9 (dCas9) binds to target regions without catalytic activity, and this property enables the design of various tools [79]. For example, the dCas9-SunTag system, formed by fusing dCas9 with a transcriptional activation domain, can significantly increase gene transcription levels; dCas9 fused with DNA methyltransferases or demethylases can add or remove methyl groups at targeted genomic locations, thereby controlling gene silencing or enhanced expression [80]. dCas9 fused with acetyltransferases can regulate histone acetylation levels, thereby modulating chromatin accessibility and gene transcription [81]. Similarly, dCas9 fused with histone methyltransferases or demethylases is also involved in regulating histone methylation levels [79]. Using these tools and epigenetic modification markers, the expression of cold-tolerance-related genes can be precisely engineered to control the quality deterioration caused by cold stress in horticultural crops. The new traits resulting from these epigenetic modifications can be stably inherited to some extent, thereby enhancing the cold stress tolerance of horticultural crops. Although most of these technologies are still in the research stage and have not yet been fully applied in the economic sector of horticultural crops, their application in plants demonstrates the feasibility of this regulatory mechanism.

### Exogenous elicitors with epigenetic effects

Beyond the targeted fine-tuning enabled by gene-editing tools, certain exogenous elicitors can modulate epigenetic modifications in horticultural crops. It can also be understood as elucidating the mechanism through which exogenous treatments mitigate CI in horticultural crops via epigenetic regulation. Specifically, these exogenous treatments are divided into physical and chemical treatments. Among physical methods, besides cold acclimation, heat treatment regulates embryogenesis in cabbage and fruit ripening in tomato by affecting dynamic DNA methylation [82, 83]. Specific acoustic wave treatments can influence histone modifications related to ripening in tomato fruit; Results show that 1 kHz acoustic wave treatment for 6 h reduces histone modifications of transcription-activating and increases histone modifications of regulators that inhibit fruit ripening, thereby suppressing tomato fruit ripening; this is achieved by affecting methylation and acetylation levels [84]. Applying acoustic waves with different intensities and durations to precisely modulate cold-responsive gene expression could be a highly promising control strategy. Among chemical treatments, the most direct are histone/DNA modification inhibitors. 5-Azacytidine, as a DNA demethylating agent, regulates the redox system and energy homeostasis by inhibiting DNA methylation during the cold storage of banana fruit, helping to control skin browning in bananas [85]. The histone H3 lysine methyltransferase inhibitor GSK343 can suppress H3K27me3 methylation during cold-induced flowering in litchi, thereby increasing the expression of related genes [29]. These inhibitors can directly regulate the methylation levels of cold-responsive genes in crops, showing great potential for controlling CI, but they have certain toxicity. Further research is needed on how to control toxicity levels to ensure they are harmless to the environment and humans. Moreover, noninhibitor applications also play a significant role in controlling CI. The application of NO enhances the expression of *PpCBF5*, *PpICE1*, and *PpMYC2* by inducing increased methylation of their promoters and demethylation of the *PpCOR* promoter, thereby improving the cold resistance of postharvest peach fruit [28]. Exogenous treatment with caffeic acid increases the H3K4me3 methylation and transcription levels of genes related to flavonoid biosynthesis, promoting flavonoid accumulation and inhibiting quality deterioration in fresh-cut pineapple at 4°C [43]. Exogenous application of glycine betaine significantly reduces the cold-induced increase in m^6^A methyltransferase gene expression and further enhances m^6^A demethylase gene expression in pepper plants, markedly suppressing CI [65]. In addition, the most widely used class of chemical treatments is plant hormones. For example, MeJA treatment inhibits DNA methylation levels in the promoters of peach fruit genomes, alleviating CI and promoting fruit quality formation [27]. Similarly, in Nanguo pear fruit stored at 0°C, MeJA treatment reduces histone acetylation levels at the promoter of the key ester aroma catabolism gene *Carboxylesterase15*, thereby suppressing its expression and alleviating the decline in aroma compounds under low-temperature stress, controlling quality loss due to chilling [86]. Melatonin treatment enhances the activity of methyl esterase and demethylase in cold-stored peach fruit, regulating DNA methylation levels of key genes involved in phenolic compound synthesis, thereby alleviating low-temperature-induced fruit browning [26]. Additionally, plant hormones such as abscisic acid and ethylene have also been found to participate in the epigenetic modification-mediated quality regulation of horticultural crops under low temperatures [87, 88]. These studies reveal new regulatory mechanisms explained from an epigenetic perspective, as well as novel strategies for fine-tuning the cold tolerance of horticultural crops by combining new mechanisms with advanced technologies.

## Future prospects of epigenetic modification in horticultural crops

Current applications of epigenetics in horticultural crops still face significant limitations. The primary challenge is that the functional mechanisms of numerous epigenetic modifications remain unclear, as detailed below:

### Histone phosphorylation

Current epigenetic research in horticultural crops primarily focuses on DNA methylation and histone methylation/acetylation, while exploration of histone phosphorylation is still insufficient. Histone phosphorylation modifications are primarily involved in transcriptional activation, DNA repair and recombination, and chromosome condensation and differentiation [89, 90]. Since phosphate groups are negatively charged and repel the negatively charged DNA, histone phosphorylation modifications typically facilitate chromatin dissociation [91]. Currently, there is a lack of research on histone phosphorylation in horticultural crops, and the limited studies in plants have focused on H3, which has numerous phosphorylation modification sites. The phosphorylation of these modification sites in response to cold stress has been demonstrated in some plants. For instance, significant histone H3 phosphorylation was observed in the metaphase chromosomes of meristematic tissues in barley, rye, and broad beans following ice-water treatment, mainly in cold-sensitive regions of localized heterochromatin [92]. Additionally, histone H3 phosphorylation is a marker of mitosis and meiosis, and cell cycle-dependent phosphorylation studies in barley and rye have shown that histone H3 phosphorylation is associated with chromosome condensation during mitosis [93]. This may be because H3 phosphorylation collaborates with other PTMs to recruit chromatin remodeling complexes, chromatin compaction factors, or condensin complexes [94, 95]. Such dynamic cooperative regulation could be a key mechanism for plants to adapt to diverse physiological and stress conditions. For instance, in *Arabidopsis* and tobacco, cold stress induces rapid phosphorylation of H3Ser10, upregulating the expression of three related transcription factors, likely due to transient acetylation of H3 and H4 in response to phosphorylation [96, 97]. Furthermore, phosphorylation of H3Ser10 in *Arabidopsis* may interfere with the methylation of H3K9 and the acetylation of H3K14 [98]. These evidences indicate that the crosstalk between histone H3 phosphorylation and other PTMs in coordinately regulating chromatin changes may be a crucial mechanism for plants to cope with cold stress, but corresponding studies are lacking in horticultural crops for further verification. Cold is a significant factor affecting plant mitosis and meiosis [92, 99], but whether H3 phosphorylation is involved in this process requires further validation and analysis. Additionally, how phosphorylation of other histones like H4 regulates plant physiological processes is also a key focus for future research.

### Histone ubiquitination

In plants, histone ubiquitination mainly targets histones H2A and H2B; mono-ubiquitination of H2A and H2B (H2Aub1/H2Bub1) is abundant and highly conserved in eukaryotes, typically not causing histone degradation but closely linked to transcriptional activity [97, 100, 101]. H2Aub1 is catalyzed by the Polycomb Repressive Complex 1-like E3 ubiquitin ligase complex and primarily mediates gene silencing during nonspecific differentiation; H2Bub1 is catalyzed by two E3 ligases, histone monoubiquitination1 (HUB1) and HUB2, and mainly mediates transcriptional activation [100, 102]. Studies have found that mono-ubiquitination of H2Bub1 is always associated with histone H3K4 or H3K36 methylation and cooperatively regulates gene expression, which may be an important mechanism for histone ubiquitination to coordinately regulate cold-responsive gene expression [103]. For instance, the absence of H2Bub1 in *Arabidopsis* alters the levels of histone H3K4me3 and H3K36me3, thereby influencing the expression of the floral repressor *FLC* [103]; another study revealed that cold stress activates *FLC* expression in *Arabidopsis* via the E3 ubiquitin ligase High Expression of Osmotically Responsive Gene 1 (HOS1) [104], but the connection between these two phenomena needs further investigation. H2Bub1 is also essential for controlling the transcription of auxin biosynthesis genes and ethylene signaling-related genes [97], which may be another pathway through which histone ubiquitination participates in the regulation of cold stress in plants. In addition, poly-ubiquitination at K48 of histones typically serves as a signal for proteasome-targeted degradation, while poly-ubiquitination at K63 affects protein–protein interactions [100]. Thus, different types of histone ubiquitination modifications may have distinct functions. Revealing the roles of different histone ubiquitination modifications in horticultural crops will help fundamentally identify methods to control quality deterioration in horticultural crops and their byproducts.

### 6mC methylation of DNA

6mC is another important methylation modification in plants, and its research in low-temperature regulation mainly focuses on *Arabidopsis* and crops. For example, cold-induced 6 mA hypermethylation in gene bodies was observed in both *Arabidopsis* and rice; while these hypermethylated genes showed functional enrichment across multiple biological processes, their correlation with transcriptional changes was highly variable, implying complex regulatory roles of 6 mA in gene expression control [105], and earlier studies found a negative correlation between 6 mA methylation and cold tolerance in rice [106]. Regrettably, the functional significance of 6 mA in cold adaptation mechanisms of horticultural species remains poorly explored.

### Other fields

Current understudied areas in horticultural crops include not only typical histone phosphorylation/ubiquitination but also glycosylation, sumoylation, and carbonylation modifications, all of which are part of the histone code. The histone code is closely related to environmental stress response ‘stress memory’. Equally critical are ncRNAs that orchestrate epigenetic reprogramming in horticultural species. Although extensive research on ncRNAs has been conducted in horticultural crops in recent years, their mechanisms in epigenetic regulation have only revealed the tip of the iceberg. Phytohormones, as key players in growth, development and stress responses of horticultural crops, require further exploration of their deep epigenetic regulatory mechanisms in quality control. Moreover, in practical applications, existing Cas9-mediated epigenetic markers may be influenced by more factors during generational transmission. There is a need to optimize conditions and further observe which epigenetic states can be stably inherited in specific contexts, as well as to develop new specific epigenetic regulatory tools for large-scale application in controlling postharvest quality deterioration. Genes related to antifreeze proteins and quality formation can be directionally induced through epigenetic modifications, thereby improving the quality and economic value of horticultural crops during low-temperature stress. Precise artificial editing for targeted regulation of plant traits will become the mainstream direction for future horticultural crop development.

## Conclusions

**Figure 5:**
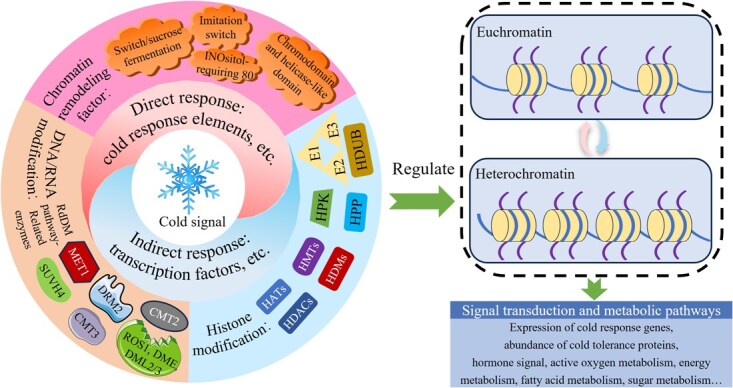
Mechanisms of epigenetic involvement in regulation of cold stress in horticultural crops.

Epigenetic regulation is the direct mechanism by which horticultural crops rapidly respond to low-temperature signals. The low-temperature signals generated by cold stress affect the activity of chromatin remodeling factors and epigenetic modification enzymes through direct or cascade regulation. These proteins modulate chromatin accessibility through multilayered epigenetic regulation, thereby mediating the phenotype and quality of horticultural crops by controlling gene expression levels (Fig. 5). Therefore, the CI phenotype essentially reflects an imbalance between chromatin openness and transcriptional activity under cold stress. Additionally, the ‘cold stress memory’ may be related to ‘long-term stress’ or the ‘histone code’. The gene editing technologies and exogenous treatments can activate this ‘stress memory’ or short-term epigenetic regulation, enabling specific gene expression regulation in response to low-temperature stress, thereby improving the quality of horticultural crops during cold stress.
